# Influence of conception and delivery mode on stress response marker *Oct4B1* and imprinted gene expression related to embryo development: A cohort study

**DOI:** 10.18502/ijrm.v19i3.8569

**Published:** 2021-03-21

**Authors:** Maria Argyraki, Socrates Katafigiotis, Theofanis Vavilis, Zoe Papadopoulou, Giorgos Tzimagiorgis, Anna-Bettina Haidich, Katerina Chatzimeletiou, Grigoris Grimbizis, Basil Tarlatzis, Maria Syrrou, Alexandros Lambropoulos

**Affiliations:** ^1^Laboratory of Genetics, 1st Department of Obstetrics and Gynecology, School of Medicine, Aristotle University of Thessaloniki, “Papageorgiou” General Hospital, Thessaloniki, Greece.; ^2^Laboratory of Biology and Genetics, School of Medicine, Aristotle University of Thessaloniki, Thessaloniki, Greece.; ^3^Laboratory of Biology, Faculty of Medicine, School of Health Sciences, University of Ioannina, Ioannina, Greece.; ^4^Laboratory of Biological Chemistry, School of Medicine, Aristotle University of Thessaloniki, Thessaloniki, Greece.; ^5^Department of Hygiene and Epidemiology, School of Medicine, Aristotle University of Thessaloniki, Thessaloniki, Greece.; ^6^Unit for Human Reproduction, 1st Department of Obstetrics and Gynecology, School of Medicine, Aristotle University of Thessaloniki, “Papageorgiou” General Hospital, Thessaloniki, Greece.

**Keywords:** Conception, Fertilization in vitro, Genomic imprinting, Fetal blood.

## Abstract

**Background:**

Recent scientific data support that the mode of conception and delivery may influence epigenetic regulation and therefore embryo development. Octamer-binding transcription factor 4-B1 *(OCT4B1)*, a novel variant of OCT4 with yet unknown biological function, is suggested to have a potential role in mediating cellular stress response. Furthermore, *Insulinlike Growth Factor 2* *(IGF2)*, *Mesoderm-specific Transcript* *(MEST) *and *paternally expressed gene 10* *(PEG10) *are genes known as imprinted and are regulated via means of epigenetic regulation. The influence of delivery mode and conception on epigenetic regulation is an active research field.

**Objective:**

Our aim was to correlate the expression level of *Oct4B1* and the expression and methylation level of *IGF2*, *MEST*, and *PEG10 *imprinted genes with the mode of delivery and conception in the umbilical cord blood of newborns.

**Materials and Methods:**

Samples of umbilical cord blood from infants born after vaginal delivery, caesarean section (CS) with the infant in cephalic position and CS due to breech position were examined. Furthermore, the investigation included infants conceived through means of assisted reproductive technology.

**Results:**

No statistically significant differences were found in mRNA expression levels between different modes of conception and delivery (p = 0.96). *Oct4B1*, *IGF2*, *MEST*, and *PEG10 *expression levels do not seem to be significantly affected by different modes of conception and delivery.

**Conclusion:**

These results indicate that the expression and methylation patterns of *Oct4B1*, *IGF2*, *MEST* and *PEG10* in umbilical cord blood are not affected by the conception and delivery mode.

## 1. Introduction

Vaginal delivery (VD) exposes infants to various stressors absent during caesarean section (CS), such as the secretion of catecholamines and cortisol, which trigger hormonal cascades in order to prepare the infant for life after birth (1). Furthermore, the artificial interventions employed during assisted reproductive technologies (ARTs), coincide with the embryos' epigenetic reprogramming, thus leading possibly to aberrant establishment and maintenance of genomic imprints and an increase in imprinting disorders (2-5). On the other hand, concerning cellular stress, the octamer-binding transcription factor 4-B1 isoform, *(Oct4B1)*, has emerged as a possible cell stress marker (6). This isoform has been found to be an emergent marker of stemness in embryonic cells and is also implicated in apoptosis and probably in cancer (7-10). In the present study, *Oct4B1* was selected to be studied because of its putative role in cellular stress response. This is the first study conducted in umbilical cord blood (UCB) concerning *Oct4B1*. Other genes susceptible to epigenetic modification upon stress exposure are *Insulin Growth Factor 2* (*IGF2*), *Mesoderm-Specific Transcript* (*MEST*), and *Paternally Expressed Gene 10* (*PEG10*). These genes affect fetal growth and their disruption is linked to metabolic disorders, cognitive impairment, low birth weight, and some types of cancer (11-13). These genes were selected to be studied because of their crucial role in fetal development, as they control embryonic and placental growth. Furthermore, these imprinted genes are responsive to different in utero environments and thus may mediate adverse environmental signals to the embryo during pregnancy. There are contradictory results in literature concerning the effect of ART on these genes. Lastly, there are no previous reports of the expression of these genes in embryos with breech presentation.

Given that various environmental factors and stressors can affect the epigenetic processes, concerns are raised about the implications of the increasing prevalence in elective CS deliveries and the use of ART. Our aim was to investigate the role of different modes of conception and delivery as possible environmental stressors on epigenetic regulation and stress response. Therefore, we evaluated the expression level of *Oct4B1* and the expression and methylation status of *IGF2*, *MEST,* and *PEG10* imprinted genes in UCB samples of newborns.

## 2. Materials and Methods

### Sampling method

In this cohort study, 40 participants were enrolled whose UCB samples were collected during the time of their delivery. The samples were subdivided into four groups that comprised of 10 participants each. The extremely strict inclusion criteria did not allow for a larger sample size to be collected. This may be a disadvantage for the statistical analysis of the results, but it reflects a very homogenous and carefully designed sample population. The groups examined were VD (control group), CS with cephalic projection of the embryo, and CS with breech embryo projection. Additionally, the fourth group consisted of infants conceived through ART and delivered through caesarian section.

The eligibility for participation included uncomplicated full-term deliveries (≥ 37 wk) of healthy parents with an existing full medical record and supervision of the pregnancy by an obstetrician. On the other hand, the exclusion criteria included positive smoking status, chronic or acute diseases, twin gestations, non-compliance to the required medical tests during pregnancy, and pregnancies with embryos presenting chromosomal or anatomical abnormalities. All biospecimens collected were done so with the participant's informed written consent after having secured permission from the Scientific Committee of Papageorgiou General Hospital of Thessaloniki, the Bioethics Committee of the School of Medicine of Aristotle University of Thessaloniki, and from the Hellenic Data Protection Authority.

### Quantitative real-time polymerase chain reaction (qRT-PCR)

Total RNA was extracted from leucocytes using TRIZOL reagent (Invitrogen, USA), according to the manufacturer's instructions. RNA samples were treated with TURBO DNA-free${}^{\mathrm{TM}}$ DNase (Ambion, Life Technologies, USA). A no-reverse transcription (no-RT) control was used for each sample to exclude any potential non-specific amplification of genomic DNA. The SuperScript${}^{\mathrm{TM}}$ First-Strand Synthesis System (Invitrogen, USA) was used to reverse transcribe total RNA with random hexamer primers. Quantitative Real-Time PCR employed TaqMan${}^{\mathrm{TM}}$ Gene Expression MasterMix reagent (Thermo Scientific, USA) and TaqMan${}^{\mathrm{TM}}$ Gene Expression Assays for evaluation of Beta-2 Microglobulin (*B2M*, endogenous control) *IGF2*, *MEST,* and *PEG10 *transcription. The primers for *Oct4B1 *were previously described (Table I). Quantification of the results was performed using the 2-ΔΔ Ct  method after normalization against *B2M*.

### Methylation-specific PCR

For DNA extraction, DNA Mini Kit (Qiagen, Netherlands) was used. DNA recovered from the aforementioned process was chemically converted using EpiTectPlus DNA Bisulfite Kit (Qiagen, Netherlands), in order to turn unmethylated cytosines to uracil for detection by methylation-specific PCR. Next, the bisulfite-treated DNA was subjected to PCR for detection of the methylated and unmethylated *MEST*, *IGF2*, and *PEG10 *alleles (Table II). Samples containing no DNA were used as a negative control, whereas for positive control, bisulfite-treated DNA from peripheral blood was used.

**Table 1:** Primers used for qRT-PCR


*Gene*	**TaqMan gene expression assay ID**	**Amplicon size**
*B2M*	Hs00984230_m1	81bp
*IGF2*	Hs00171254_m1	93bp
*MEST*	Hs01040913_g1	93bp
*PEG10*	Hs01122880_m1	87bp
*Oct4B1*	F: 5' GGGTTCTATTTGGTGGGTTCC 3' R: 5' TCCCTCTCCCTACTCCTCTTCA 3'	128bp
*Primers for Beta-2 Microglobulin* *(B2M)*, *Insulin Growth Factor 2* *(IGF2)*, *Mesoderm-Specific Transcript* *(MEST), *and *Paternally Expressed Gene 10* *(PEG10) *were commercially available from Thermo Fisher Scientific. Primers for *Oct4B1 *according to Farashahi Yazd E and colleagues (7). B2M: 81bp, IGF2: 93bp, MEST: 93bp, PEG10: 87bp, OCT4B1: 128bp		

**Table 2:** Sequence and characteristics of methylation-specific PCR primers for *MEST-, PEG10-*, and *IGF2-*imprinted genes


**Gene name**	**Sequence (5'→3')**	**Tm (°C)**	**GC content**	**Product**
*MEST* **meth/prF**	TAGTTGCGTTTCGTAAGGTAGTGTC (25)	61.3	44.0%	291bp
*MEST* **meth/prR**	ACACAATCCTCCGCTCGCCTA (21)	61.8	57.1%	
*MEST* **unmeth/prF**	GTGGTAGTTGTGTTTTGTAAGTGTAGTGTT (30)	62.7	36.7%	296bp
*MEST* **unmeth/prR**	CACACAATCCTCCACTCACCTACA (24)	62.7	50.0%	
*PEG10* **meth/prF**	AGGTTCGTTGAGCGGGTTGTCGTTCG (26)	64.7	57.7%	184bp
*PEG10* **meth/prR**	TCCCGATAAACACGAAAAATAAACG (25)	54.1	36.0%	
*PEG10* **unmeth/prF**	AGGTTTGTTGAGTGGGTTGTTGTTTG (26)	58.4	42.3%	184bp
*PEG10* **unmeth/prR**	TCCCAATAAACACAAAAAATAAACA (25)	50.2	24.0%	
*IGF2* **meth/prF**	TTAATTGGGGTTCGTTCG (18)	51.4	44.4%	118bp
*IGF2* **meth/prR**	CCCGACCTAAAAATCTAATACGA (23)	57.1	39.1%	
*IGF2* **unmeth/prF**	GGTTTGTTTGTGGAAATGTTTT (22)	52.8	31.8%	110bp
*IGF2* **unmeth/prR**	CCCAACCTAAAAATCTAATACAA (23)	53.5	30.4%	
Primers *MEST*: Kosaki *et al* (14). Primers *IGF2*: Dimitriadou *et al* (15). Primers *PEG10*: Designed for the first CpG-island of the gene. TM: Meth 57°C/unmeth 60°C, GC: 44%, Meth: Methylated allele, Unmeth: Unmethylated allele, prF: Primer forward, prR: Primer reverse, MEST: Mesoderm-specific transcript, PEG: Paternally expressed gene 10, IGF2: Insulin-like Growth Factor 2				

### Ethical considerations

All procedures performed in studies involving human participants were in accordance with the ethical standards of the Scientific Committee of Papa Georgiou General Hospital of Thessaloniki (reference number: 157/27.06.12), the Bioethics Committee of the School of Medicine of Aristotle University of Thessaloniki (reference number: 1/8-11-2012), and of the Hellenic Data Protection Authority (reference number: 1145) and with the 1964 Helsinki Declaration and its later amendments or comparable ethical standards. In addition, a written informed consent was obtained from individual participants included in the study.

### Statistical analysis

Data were analyzed using the IBM SPSS Statistics version.24 (SPSS Inc., Chicago, Illinois, USA) program. Demographic data were normally distributed (based on Shapiro test for normality) and one-way ANOVA-test was used for quantitative variables (maternal age, maternal weight, gestational week, and birth weight) and χ${}^{2}$-test for qualitative variables (gender of the newborns and ratio of primiparae women). The gene expression results were evaluated through non-parametrical tests due to the small sample size, and more specifically the Kruskal-Wallis test was employed to compare *Oct4B1*, *IGF2*, *MEST*, and *PEG10* expression between the four groups. Results were deemed significant only when p < 0.05.

## 3. Results

**Figure 1:**
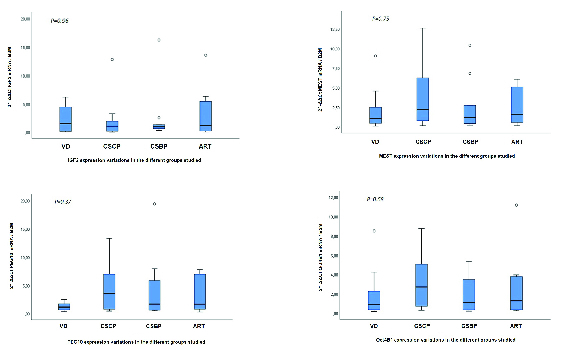
Expression of *IGF2*, *MEST, PEG10*, and *Oct4B1 *genes in UCB from infants derived through vaginal delivery (VD), caesarean section with cephalic embryo projection (CSCP), caesarean section with breech embryo projection (CSBP), and assisted reproduction technologies (ARTs). Each group consists of 10 samples. Samples were normalized against *B2M* and compared with the control group. Median values ± SD presented.

**Figure 2:**
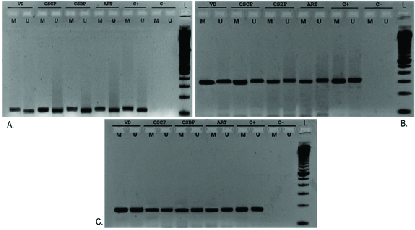
Methylation analysis of *IGF2* (A), *MEST* (B), and *PEG10 *(C), respectively, for some samples of all groups, including positive (C+) and negative (C-) controls. VD: Vaginal delivery, CSCP: Caesarean section with cephalic embryo projection, CSBP: Caesarean section with breech embryo projection, ART: Assisted reproduction technologies, L: Ladder 100bp, M: Methylated allele, U: Unmethylated allele. PCR product sizes: *IGF2*: 118bp (M), 110bp (U), *MEST*: 291 (M), 296bp (U), *PEG10*: 184bp (M), 184bp (U).

As mentioned above, 40 participants were enrolled whose UCB samples were collected during the time of their delivery (Table III). Quantitative Real-time PCR revealed no statistically significant differences at the transcriptional level between the VD, CS, and ART groups for *Oct4B1* (p = 0.58), *IGF2* (p = 0.96), *MEST* (p = 0.75), and *PEG10* (p = 0.37) (Figure 1). *PEG10* exhibited a non-significant trend for up regulation (p = 0.089) in the group of cephalic presentation CS compared to the VD group. Methylation-Specific PCR products were subjected to gel electrophoresis in order to validate the presence of both methylated and unmethylated alleles for *IGF2*, *MEST*, and *PEG10* for all 40 samples (Figure 2). Methylation analysis was not possible for *Oct4B1* due to the existence of alternative promoters for this isoform, incommoding primer design (16). Methylation status is in agreement with non-statistical differences in expression.

**Table 3:** Demographic data of the enrolled participants


	**Vaginal delivery**	**Caesarean section with cephalic embryo projection**	**Caesarean section with breech embryo projection**	**Assisted reproduction technologies**	**P-value**
Maternal age*	32.3 ± 3.37	33.6 ± 3.72	31 ± 4.35	35.2 ± 3.12	0.084
Maternal weight*	74.6 ± 4.97	73.3 ± 4.52	74.3 ± 5.05	73.4 ± 3.44	0.89
Gestational week*	38.7 ± 1.05	37.9 ± 0.65	38.3 ± 1.06	38 ± 0.74	0.142
**Primiparae women**	50%	40%	60%	70%	0.57${}^{+}$
Birthweight* (kg)	3.21 ± 0.38	3.22 ± 0.39	3.34 ± 0.44	3.02 ± 0.18	0.289
**Gender (male)**	40%	50%	40%	60%	0.776${}^{+}$
*Data presented as Mean ± SD. One-way ANOVA, ${}^{+}$x${}^{2}$-test for qualitative variables					

## 4. Discussion

It is assumed that imprinted genes can serve as environmental sensors, since they can be responsive to environmental stressors and alter their expression and/or methylation during specific developmental stages.

In the current study, we investigated the effect of the conception and delivery mode, which are considered potential environmental stressors, on the expression and methylation levels of three imprinted genes. Furthermore, we evaluated their effect on the expression of *Oct4B1* isoform, a potential stress-response marker.

In our study, it appears that different modes of conception and delivery do not alter significantly the mRNA expression levels for *Oct4B1*, *IGF2*, *MEST*, and *PEG10* in UCB. This could be attributed not only to the small sample size available, but also to the extremely strict inclusion criteria, excluding any kind of fetal abnormality, complicated pregnancies, mothers with diagnosed health conditions, and unsupervised pregnancies.

To date, to the best of our knowledge, no other reports exist in literature investigating the expression levels of the *Oct4B1 *isoform in UCB. *Oct4B1 *is not only a newly discovered variant of Oct4 family that bestows stemness qualities in cell expressing it, but also seems to serve an anti-apoptotic function in a multitude of cancer cell lines (9, 10, 17, 18). Furthermore, *Oct4B1* warrants further investigation as a regulator of cellular stress response since it plays a role in the regulation of HSP40 family of chaperones (17). The implications of this regulation are not clear at the moment, but it is possible that *Oct4B1* might modulate globally the stress response since the HSP40 family constitutes the most diverse and largest category of HSPs and its members are known to interact with other actively researched HSP families such as HSP70 and HSP90 (19). Different stress conditions during CS and ART could probably result in alterations in *Oct4B1* expression levels. However, this was not observed in our study, indicating that the stress variations during different conception and delivery modes are not capable of affecting *Oct4B1* expression.

Furthermore, this is the first study on the expression and methylation of those genes that correlate them to embryo presentation during CS, either cephalic or breech. Such a distinction is of importance since the positioning of the embryo can indicate maturity prior to delivery and either excessive or suboptimal levels of stress could possibly interfere with methylation and future health outcomes. Interestingly, in our study, there was a trend for increased expression of *PEG10* in the CSCP group, a finding that warrants further investigation in broader sample studies. As far as the ART group is concerned, our results are consistent with previous studies, indicating stability in the expression and DNA methylation levels of the aforementioned imprinted genes. However, there are other studies reporting altered DNA methylation and expression levels of these genes after ART treatments. These contradictory results can be attributed to different ART protocols, variation in sample size, sample homogeneity, and different analysis methods.

Furthermore, there are also other mechanisms that maintain imprints, such as histone modifications and long non-coding RNAs, which should also be considered as a possible explanation to the variation of results found between studies. It remains also unclear whether the parental underlying sub-fertility Per se can act as the causal factor of the higher complication prevalence observed in the results of some reports of ART-conceived children. Lastly, the existence of an imprinted gene network could also explain the heterogeneity of the reported results. This theory was first described in animal reports (20, 21), but many scientists believe that it also refers to humans (22-24). According to this theory, the imprinted gene network includes hundreds of imprinted genes with similar roles controlling essential functions, such as fetal nutrition and growth. Thus, the inappropriate function of some imprinted genes can be compensated by the expression of others with similar role.

All evidence shows that it is necessary to understand the health risks and the underlying molecular mechanisms of ART interventions in order to increase the safety of these techniques. Thus, the contradictory literature results call for caution and for larger studies. The current study is exploratory in nature, utilizing well-characterized groups as far as the medical history of the participants and the medical supervision during pregnancy is concerned. It can be considered though as a starting point for more resolving the role of imprinted genes and *Oct4B1* isoform on the regulation of human reproduction. Due to the limitation of the sample size, further studies are needed to evaluate these results.

## 5. Conclusion

The results indicate that different modes of conception and delivery do not affect the mRNA expression levels of *Oct4B1*, *IGF2*, *MEST*, and *PEG10* in UCB. These results are the first reported in cephalic and breech presentation CS concerning these specific genes. Additionally, methylation analysis revealed the presence of both methylated and unmethylated alleles for the above aforementioned imprinted genes, with no methylation differences reported between groups.

Methylation status of all genes is in consistence with non-statistical differences in expression. Methylation analysis was not possible for *Oct4B1 *due to the existence of unknown alternative promoters for this isoform. While our study found no effect of the mode of conception and delivery on the expression of *Oct4B1*, *IGF2*, *MEST*, and *PEG10* in UCB, the expression of the genes in question should be investigated further. As far as the ART group is concerned, our results are consistent with some previous reports.

However, there are other studies reporting altered DNA methylation and expression of these genes after ART manipulations. These contradictory literature results call for caution and for larger studies. Differences may become apparent utilizing larger sample sizes, keeping though in mind that excellent characterization of the groups examined (medical history, supervision, etc.) should not be compromised for achieving the desired sample size. This would ensure that the observed effect can be strongly correlated to the mode of conception and delivery, rather than confounding variables.

##  Conflict of Interest

During the recent five-years, Basil C. Tarlatzis has received honoraria, fees, or grant support from the following organizations (in alphabetic order): DIAFERT, European Union Social Fund and the Greek Ministry of Education, European Union and the Greek General Secretariat of Research and Technology, Ferring, IBSA, MERCK, MSD, Ova science, Roche Diagnostics. The other authors declare no conflict of interest.
